# Supplementary material for: Protein embeddings predict binding residues in disordered regions
Source: Sci Rep. 2024 Jun 12;14:13566. doi: 10.1038/s41598-024-64211-4 (PMC11169622; doi:10.1038/s41598-024-64211-4)
Supplement: Supplementary file 1 — Supplementary Information. [file 41598_2024_64211_MOESM1_ESM.pdf]

Supporting online material
for:

Protein Embeddings Predict Binding Residues in Disordered Regions

Laura R. Jahn, Céline Marquet, Michael Heinzinger & Burkhard Rost

Table of Contents for Supporting Online Material

TABLE OF CONTENTS FOR SUPPORTING ONLINE MATERIAL 1

SHORT DESCRIPTION OF SUPPORTING ONLINE MATERIAL 1

MATERIAL 3

Supplementary Fig. SOM_F1: ROC curves on the validation set. 3
Supplementary Fig. SOM_F2: Performance on the validation set. 4
Supplementary Fig. SOM_F3: Training curves of all models. 5
Supplementary Fig. SOM_F4: Precision and recall at different cutoffs. 6
Supplementary Fig. SOM_F5: Length distribution of binding regions. 7
Supplementary Fig. SOM_F6: Length distribution of binding regions. 8
Supplementary Fig. SOM_F7: Prediction Profile of IDBindT5 for test set. 9
Supplementary Fig. SOM_F8: Feature Importance of IDBindT5. 10
Supplementary Table SOM_T1: Composition of the final data set. * 11
Supplementary Table SOM_T2: Model parameters. * 12
Supplementary Table SOM_T3: Performance assessment on the validation set. * 13
Supplementary Table SOM_T4: Performance assessment on the test set. * 14
Supplementary Table SOM_T5: IDBindT5 performance inputting predicted disorder* 15
Supplementary Table SOM_T6: Performance of disorder predictors on CAID2's binding dataset * 16
Supplementary Table SOM_T7: Welch's t-test applied to models on the test set. * 17
Supplementary Table SOM_T8: Speed assessment * 18

REFERENCES FOR SUPPORTING ONLINE MATERIAL 18

Short description of Supporting Online Material

Figures SOM_F1 – SOM_F4 display ROC (receiver operating characteristic) curves of all trained models, the models' performance on the validation set, the training curve of each model's best cross validation (CV)-fold and their precision-recall curve, respectively. The second plot SOM_F2 was used for deciding on the final predictor. Figures SOM_F5 – SOM_F7 explore IDBindT5's predictions on the test set in terms of binding region continuity and a prediction profile. Figure SOM_F8 depicts the feature weights of the model's input layer. Table SOM_T1 lists the number of proteins and residues of each data set. Table

SOM_T2 provides the parameters for training the models. Tables SOM_T3 and SOM_T4 contain all performance assessments that were created for the models. Table SOM_T5 shows the test set performance of two disorder predictors, which were used as input to IDBindT5 instead of experimental annotation (see Table SOM_T4). Table SOM_T6 contains performance assessments on the CAID2 binding benchmark set. Table SOM_T7 gives the results of Welch's t-test applied to our final model and two state-of-the-art methods. Lastly, Table SOM_T8 provides runtimes of IDBindT5 and SOTA models under different conditions.

Material

Supplementary Fig. SOM_F1: ROC curves on the validation set.

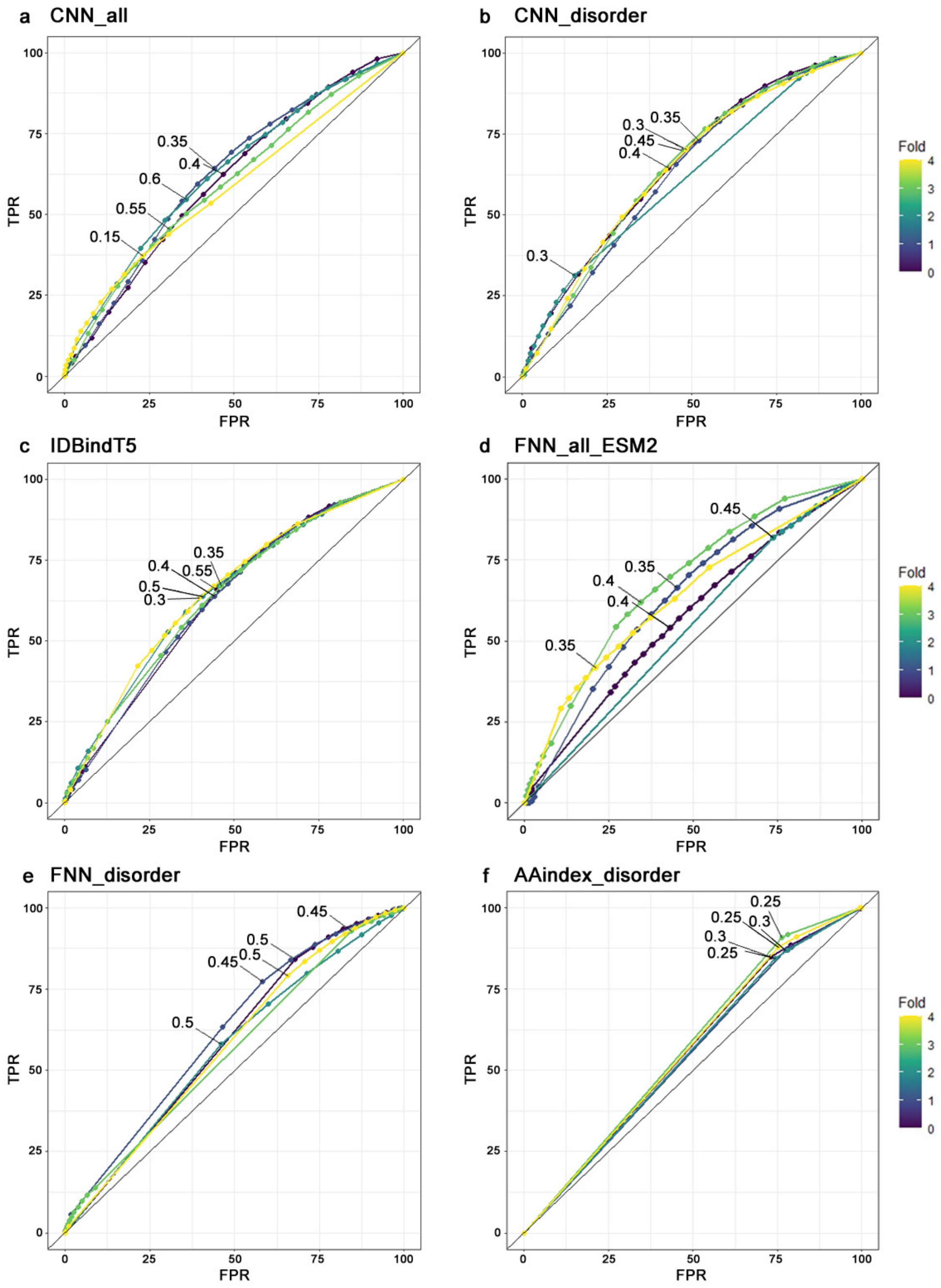

Supplementary Fig. SOM_F1: ROC curves on the validation set. These ROC curves were used to determine the optimal cut off for classifying a residue as binding (output probability of the model > cut off) or non-binding (output probability ≤ cut off). The aim was to achieve a good precision while avoiding a large drop in recall. The chosen cut-offs are annotated for each model and CV-fold.

Supplementary Fig. SOM_F2: Performance on the validation set.

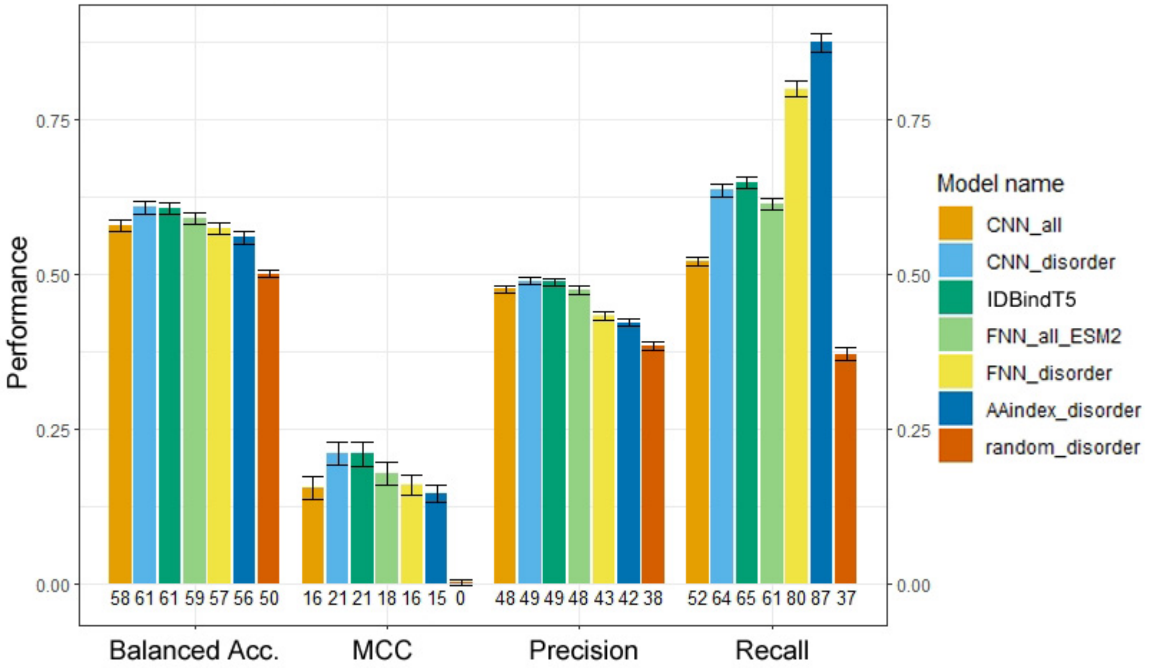

Supplementary Fig. SOM_F2: Performance on the validation set. Average performance and standard error of the six most relevant predictors and a random baseline predictor for binding regions on the validation set. The random predictor only considers the class imbalance in the disordered training set for its predictions. The performance is captured by the four per-residue measures: balanced accuracy (Eqn. 5), MCC (Eqn. 7), precision (Eqn. 1) and recall (Eqn. 2) (definitions see *Methods*). The numbers below each performance bar show the respective performance times 100, rounded to the closest integer. All models performed significantly better than the random baseline in all aspects, but they suffer from low precision. *FNN_all_ESM2*, which applies the *IDBINDT5* architecture to ESM-2 [1] embeddings, is significantly worse than model *IDBINDT5* in the aspects of precision and recall. This suggests that ProtT5 might be better suited for predicting disordered residue features than ESM-2. The simplistic model *AAindex_disorder*, which represents a baseline for models based on AA propensities only, can compete with *CNN_all* and *FNN_disorder*, but is outperformed by *CNN_disorder*, *IDBINDT5* and *FNN_all_ESM2*. We cannot see a trend in performance that would lead us to favor one architecture or training residue subset over another. However, based on predictive performance and the observation that our FNNs are faster than our CNNs, *IDBINDT5* was chosen as final predictor. More performance measures and exact values can be found in Table SOM_T3.

Supplementary Fig. SOM_F3: Training curves of all models.

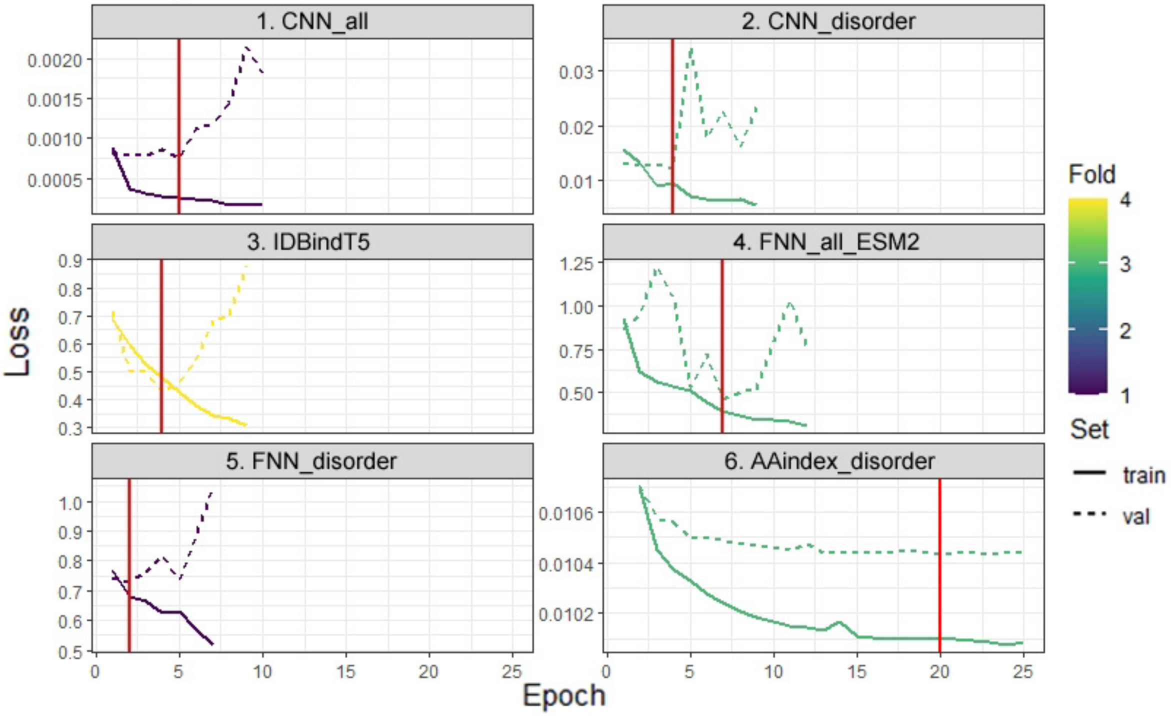

Supplementary Fig. SOM_F3: Training curves of all models. Loss on training and validation set, monitored for each epoch during training of the best CV-folds of the six relevant models. The red vertical line indicates which version was saved via early stopping with patience of five epochs. The final model, *IDBindT5*, trained for only nine epochs before early stopping saved the model of epoch four. All models trained on protein embeddings only train for a short while, whereas the model based on biochemical and -physical features, *AAindex_disorder*, trains for a much longer time of 25 epochs. We suspect that this difference might be caused by the rich information content of the embeddings, meaning the ML model only needed to filter for a few relevant features and weigh them accordingly. This has also been observed for other models trained on ProtT5 embeddings, such as bindEmbed21DL [2], TMbed [3], VESPA [4] and EMBER2 [5].

Supplementary Fig. SOM_F4: Precision and recall at different cutoffs.

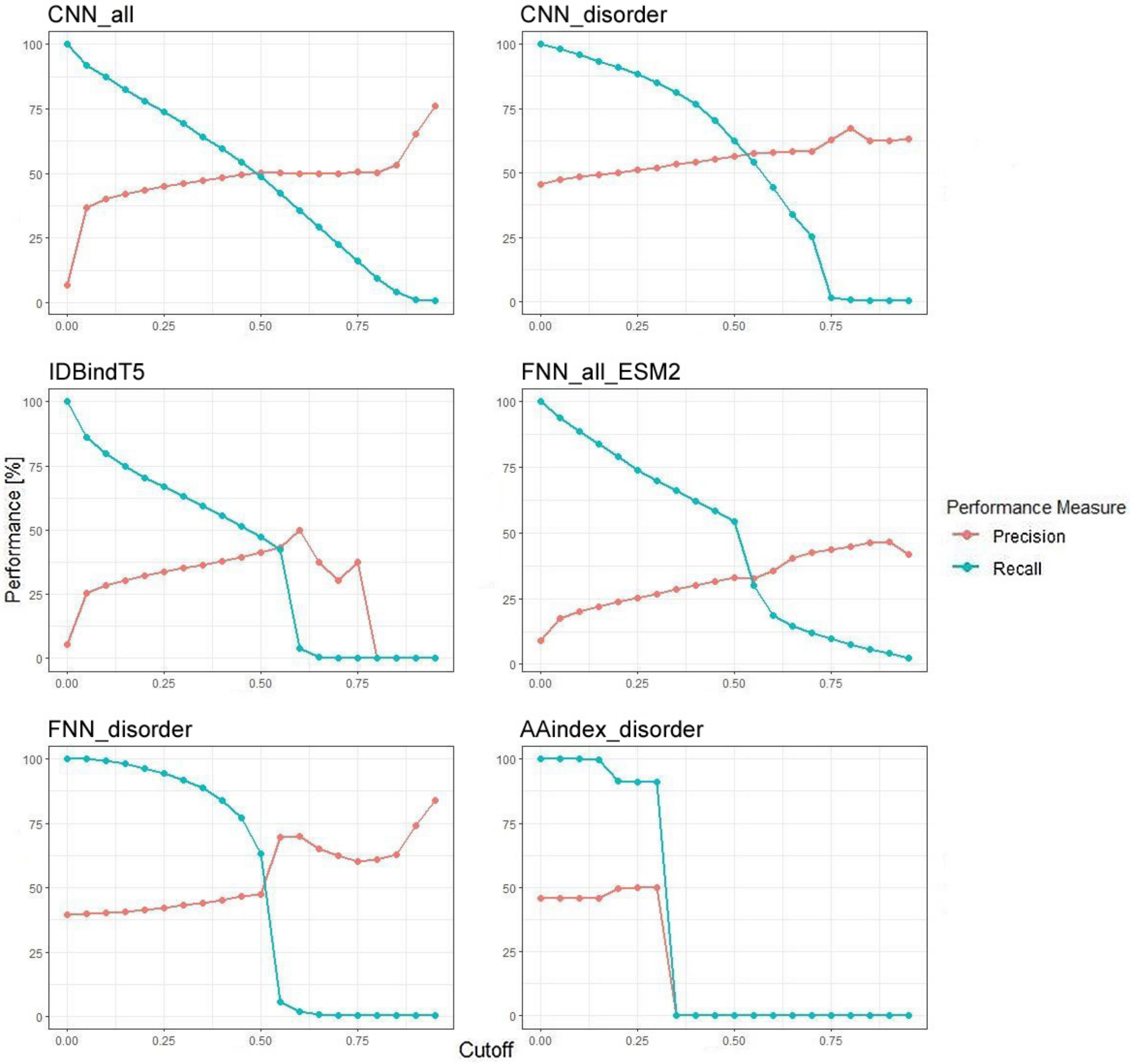

Supplementary Fig. SOM_F4: Precision and recall at different cut offs. Precision (Eqn. 1) and recall (Eqn. 2) change across different cut offs for the classification as binding or non-binding on the validation set. The x-axis gives the raw output of the model for a prediction, a.k.a. the prediction strength. The y-axis gives the average performance at the respective cut off. All curves are cumulative, i.e., the metric of all residues predicted with a value ≥ cut off is as shown on the y-axis. High cut offs correspond to more reliable binding predictions and lower cut offs lead to more precise non-binding predictions. For the optimal balanced performance, we chose *IDBindT5* at cut off 0.3 as final model. However, if a large dataset shall be scanned for few, but higher-confidence binding predictions, we recommend using *FNN_disorder* at cut off 0.55, instead.

Supplementary Fig. SOM_F5: Length distribution of binding regions.

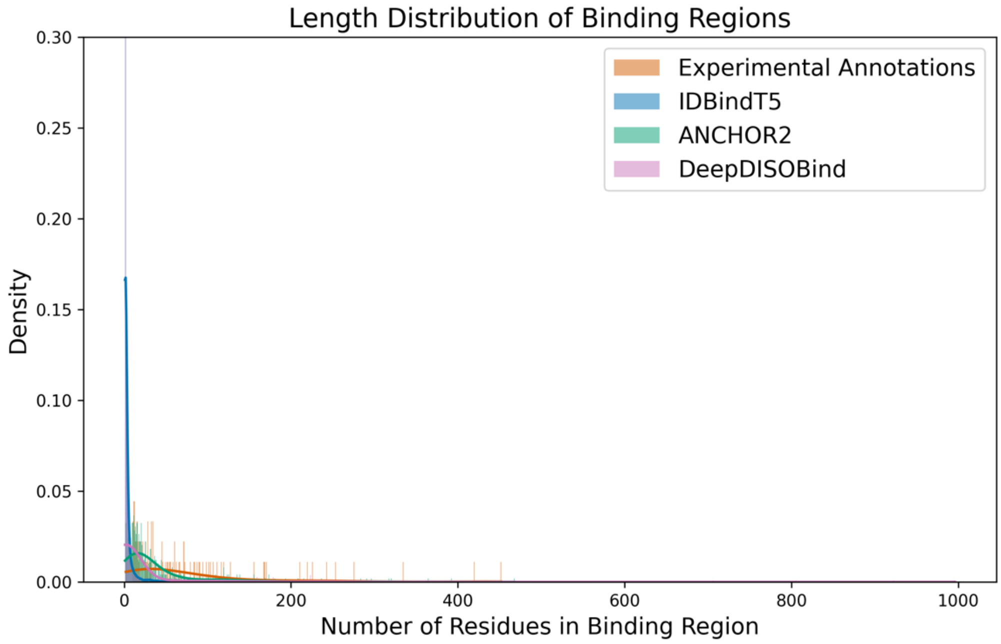

Supplementary Fig. SOM_F5: Length distribution of binding regions. The histogram shows the distribution of lengths of binding regions in for experimental and predicted values of the test set with Experimental Annotations in orange, IDBindT5 in blue, ANCHOR2 [6] in green, and DeepDISOBind [7] in pink. The x-axis represents the number of residues in the binding region, while the y-axis represents the density of occurrences. Each dataset represents the lengths of stretches of binding residues, where a value of 1 indicates a binding residue and 0 indicates a non-binding residue. Binding regions are identified as contiguous stretches of residues labelled as binding (1).

Supplementary Fig. SOM_F6: Length distribution of binding regions.

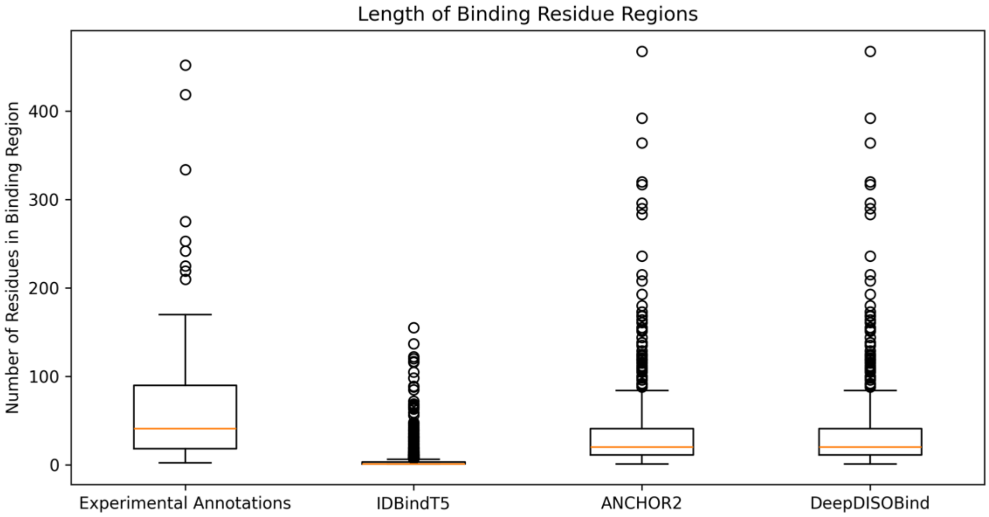

Supplementary Fig. SOM_F6: Length distribution of binding regions. The box plot depicts the distribution of binding residue region lengths in the test set for experimental annotations, IDBindT5, ANCHOR2 [6], and DeepDISOBind [7] predictions (from left to right). Each boxplot comprises a box representing the interquartile range (IQR) with the median marked by a horizontal line within the box, whiskers extending to the minimum and maximum values within 1.5 times the IQR from the quartiles, and individual data points beyond the whiskers indicating outliers. The x-axis labels specify the source of the annotations, while the y-axis quantifies the number of residues in the binding regions. This visualization facilitates comparisons between the datasets regarding measures of central tendency, spread, and potential anomalies.

Supplementary Fig. SOM_F7: Prediction Profile of IDBindT5 for test set.

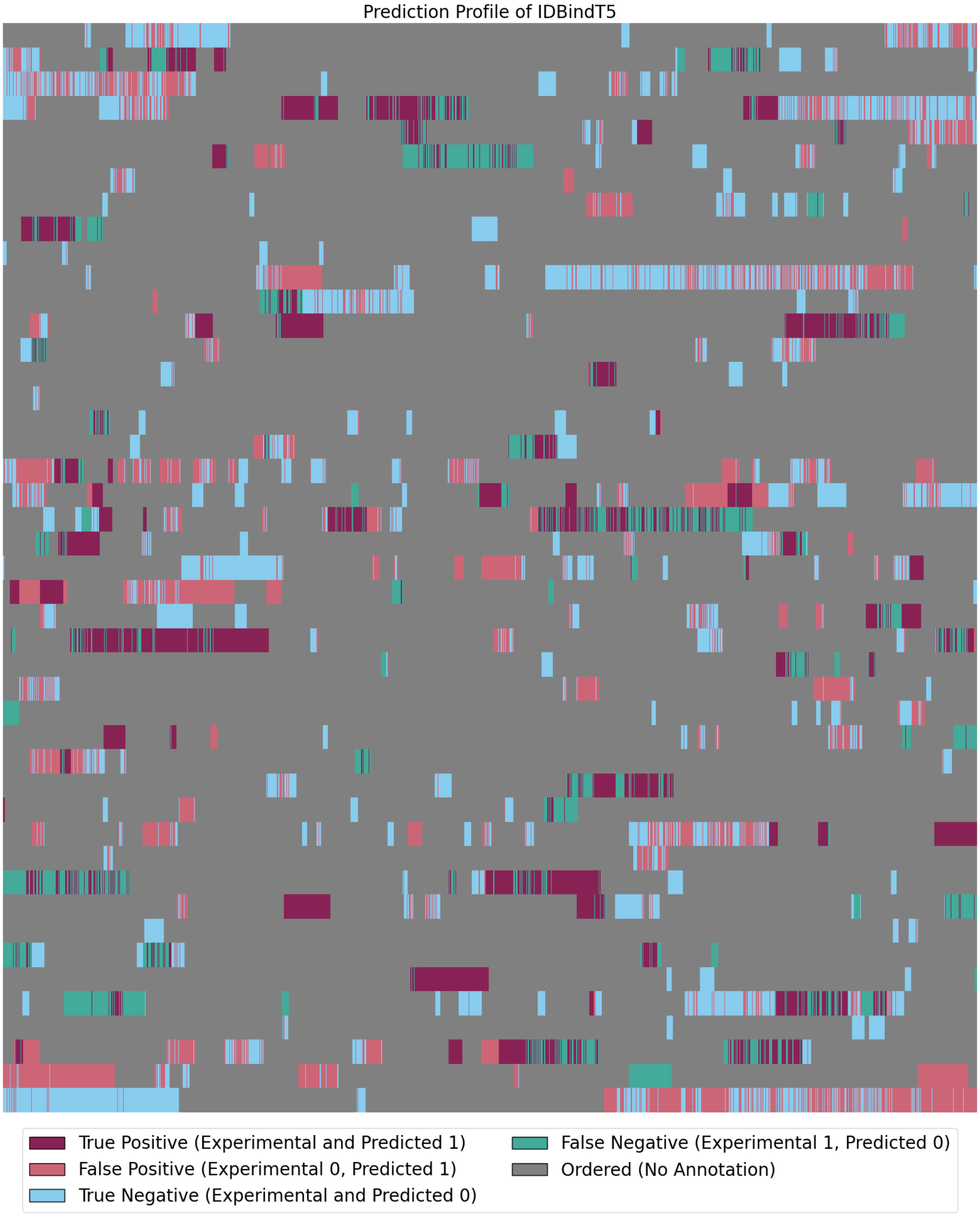

Supplementary Fig. SOM_F7: Prediction Profile of IDBindT5 for test set. The prediction profile for IDBindT5 shows all 92,373 of 195 proteins in the test set concatenated. Each pixel is colored by one of the following labels: “True Positive” with both experimental and predicted labeled binding in dark red, “False Positive” with experimental labeled non-

binding and predicted labeled binding in light red, “True Negative” with both experimental and predicted labeled non-binding in blue, “False Negative” with experimental labeled binding and predicted labeled non-binding in green, or lastly ”Ordered” residues with no annotation in grey.

Supplementary Fig. SOM_F8: Feature Importance of IDBindT5.

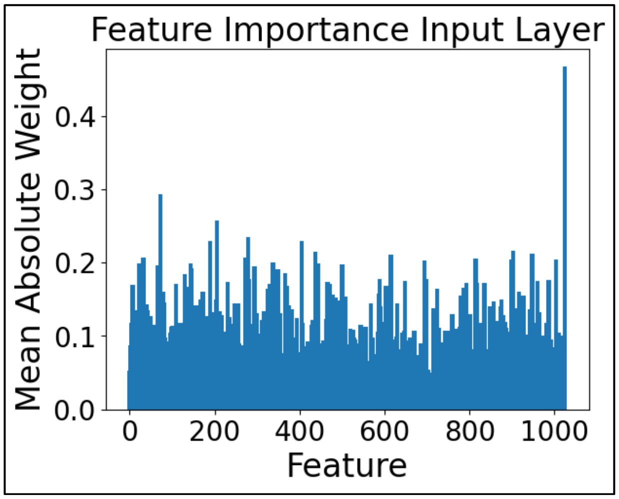

Supplementary Fig. SOM_F8: Feature Importance of IDBindT5. IDBindT5 is a feedforward neural network with one hidden layer taking a vector of length 1025 as input (ProtT5 embeddings [5] length 1024 and binary (dis)order annotation). The plot shows the feature importance as mean absolute weight of the input layer. The last layer with disorder annotation has the highest mean absolute weight, while the pLM embedding layer weights are more evenly distributed.

Supplementary Table SOM_T1: Composition of the final data set. *

Set	Subset of residues	Binding proteins	Non-binding proteins	Binding residues	Non-binding residues
**Training Set**	All	697	1,083	64,228	896,569
	IDPRs only				101,381
CV Set 0	All	139	223	10,235	165,230
	IDPRs only				20,008
CV Set 1	All	153	214	14,248	193,846
	IDPRs only				21,698
CV Set 2	All	140	196	10,386	155,712
	IDPRs only				18,665
CV Set 3	All	143	225	17,906	186,048
	IDPRs only				21,390
CV Set 4	All	122	225	11,453	195,733
	IDPRs only				19,620
**Test Set**	All	80	115	6,657	85,716
	IDPRs only				11,644

* Number of proteins and residues in their respective data sets after redundancy reduction, as described in *Methods - Data set*. The rows *CV Set 0 – 4* represent the five distinct cross validation subsets of the training set. The FASTA files can be found in the Github repository (https://github.com/jahnl/binding_in_disorder/tree/main/dataset/MobiDB_dataset_2).

Supplementary Table SOM_T2: Model parameters. *

Parameter	1. CNN_all	2. CNN_disorder	3. IDBindT5
**Input**	ProtT5 embeddings + disorder feature	ProtT5 embeddings	ProtT5 embeddings + disorder feature
**Balancing method**	Oversampling positive proteins	Undersampling negative proteins	Undersampling negative residues **
**Residue subset**	All	IDPRs only	All
**Architecture**	CNN	CNN	FNN
**# Layers**	5	5	3
**Kernel size**	5	5	-
**Dropout**	0.0	0.0	0.0
**Batch size**	1 protein	1 protein	512 residues
**Learning rate**	0.002	0.002	0.01
**Patience**	5	5	5

Parameter	4. FNN_all_ESM2	5. FNN_disorder	6. AAindex_disorder
**Input**	ESM-2 embeddings + disorder feature	ProtT5 Embeddings	AAindex1
**Balancing method**	Undersampling negative residues **	Undersampling negative residues **	Undersampling negative proteins
**Residue subset**	All	IDPRs only	IDPRs only
**Architecture**	FNN	FNN	CNN
**# Layers**	3	3	5
**Kernel size**	-	-	5
**Dropout**	0.0	0.0	0.0
**Batch size**	512 residues	512 residues	1 protein
**Learning rate**	0.01	0.01	0.002
**Patience**	5	5	5

* Parameters of the most relevant models. Models 1-3 and 5 perform best on the validation set in their respective category, which are: CNNs on all residues, FNNs on all residues, CNNs on disordered residues only, FNNs on disordered residues only. Model 5 is trained on ESM-2 [1] instead of ProtT5 [8] embeddings and applies the parameters of the *IDBindT5* model. The last model is trained on AAindex1 [9] features and adapts the parameters of the *CNN_disorder* model. An AAindex-model trained on all residues

was tried out as well, however, it did not learn anything useful for binding region prediction.

** Undersampling negative residues describes a balancing method, where negative residues within and outside of IDPRs are undersampled with different ratios, so that each subset reaches the same abundance as the positive residues.

Supplementary Table SOM_T3: Performance assessment on the validation set. *

Model	1. CNN_all	2. CNN_disorder	3. IDBindT5
**Precision**	47.7 ± 0.6%	**48.9 ± 0.6%**	**48.9 ± 0.6%**
**Recall**	52.1 ± 0.8%	63.6 ± 0.9%	64.8 ± 1.0%
**Neg. Precision**	67.7 ± 0.9%	71.6 ± 0.9%	71.6 ± 0.8%
**Neg. Recall**	**63.6 ± 0.8%**	58.0 ± 0.9%	56.7 ± 0.8%
**Balanced Acc.**	57.9 ± 0.9%	**60.8 ± 0.9%**	**60.8 ± 0.9%**
**F1 Score**	0.498 ± 0.009	0.553 ± 0.009	0.557 ± 0.009
**MCC**	0.156 ± 0.019	**0.211 ± 0.018**	0.210 ± 0.019

Model	4. FNN_all_ESM2	5. FNN_disorder	6. AAindex_disorder	Random_disorder
**Precision**	47.5 ± 0.6%	43.3 ± 0.7%	42.3 ± 0.6%	38.5 ± 0.7%
**Recall**	61.4 ± 0.8%	80.0 ± 1.3%	**87.5 ± 1.5%**	37.2 ± 1.1%
**Neg. Precision**	69.9 ± 0.9%	73.7 ± 0.9%	**75.6 ± 1.2%**	61.7 ± 0.7%
**Neg. Recall**	56.9 ± 0.8%	35.0 ± 0.8%	24.5 ± 1.0%	63.1 ± 0.9%
**Balanced Acc.**	59.2 ± 1.0%	57.5 ± 0.9%	56.0 ± 1.0%	50.1 ± 0.6%
**F1 Score**	0.536 ± 0.008	0.562 ± 0.009	**0.570 ± 0.010**	0.378 ± 0.006
**MCC**	0.179 ± 0.019	0.160 ± 0.016	0.146 ± 0.014	0.002 ± 0.004

* Performance assessment of the most relevant predictors in comparison to each other and a random baseline on the IDPRs in the validation set. The performance measures were calculated according to the definition in the *Methods* chapter (Eqn. 1-8) and rounded to three decimal points. The number after the ± refers to the 95% confidence interval of the standard error. The numerically best performances per metric are printed in bold.

Supplementary Table SOM_T4: Performance assessment on the test set. *

Model	1. CNN_all	2. CNN_ disorder	3. IDBindT5	4. FNN_all_ ESM2	5. FNN_ disorder
**Precision**	42.2 ± 1.9%	40.1 ± 2.1%	**43.2 ± 1.9%**	36.9 ± 2.5%	39.5 ± 2.1%
**Recall**	61.0 ± 3.3%	64.8 ± 3.0%	58.7 ± 2.6%	48.4 ± 2.5%	72.5 ± 3.5%
**Neg. Precision**	70.1 ± 2.7%	69.0 ± 2.9%	70.2 ± 2.8%	64.1 ± 2.6%	68.6 ± 2.9%
**Neg. Recall**	52.4 ± 2.4%	44.7 ± 2.5%	55.8 ± 2.5%	52.7 ± 2.2%	35.1 ± 2.5%
**Balanced Acc.**	56.7 ± 2.8%	54.8 ± 2.7%	57.2 ± 3.6%	50.6 ± 2.8%	53.8 ± 2.6%
**F1 Score**	0.499 ± 0.026	0.496 ± 0.027	0.498 ± 0.027	0.419 ± 0.028	0.511 ± 0.030
**MCC**	0.128 ± 0.053	0.093 ± 0.053	0.139 ± 0.070	0.011 ± 0.055	0.078 ± 0.054

Model	Consensus of models 2. and 3.	6. AAindex_ disorder	Random_ disorder	ANCHOR2 [6]	DeepDISO-Bind [7]
**Precision**	43.1 ± 1.8%	39.0 ± 2.0%	36.3 ± 2.3%	39.5 ± 1.6%	42.2 ± 2.4%
**Recall**	60.6 ± 2.8%	**86.1 ± 5.0%**	36.2 ± 2.2%	45.0 ± 2.6%	51.4 ± 3.2%
**Neg. Precision**	70.6 ± 2.9%	**74.3 ± 3.6%**	62.8 ± 2.4%	65.2 ± 2.7%	70.6 ± 3.3%
**Neg. Recall**	54.2 ± 2.3%	22.9 ± 3.0%	62.9 ± 3.4%	59.9 ± 2.4%	62.3 ± 3.4%
**Balanced Acc.**	**57.4 ± 3.0%**	54.5 ± 2.8%	49.6 ± 2.2%	52.4 ± 2.7%	56.9 ± 5.6%
**F1 Score**	0.503 ± 0.030	**0.537 ± 0.033**	0.363 ± 0.021	0.421 ± 0.036	0.463 ± 0.045
**MCC**	**0.142 ± 0.065**	0.110 ± 0.036	-0.009 ± 0.019	0.048 ± 0.060	0.133 ± 0.120

* Performance assessment on the IDPRs in the test set. The most relevant predictors are compared to each other, a random baseline, the established tools ANCHOR2 [6] and DeepDISOBind [7], and IDBindT5 on several predicted disorder labels. The performance measures were calculated according to the definition in the *Methods* chapter (Eqn. 1-8) and rounded to three decimal points. The number after the ± refers to the 95% confidence interval of the standard error. The numerically best performances per metric in bold.

Supplementary Table SOM_T5: IDBindT5 performance inputting predicted disorder*

Disorder Predictor	SETH [10]	Alphafold-disorder [11]
**Precision**	37.3%	40.2%
**Recall**	66.5%	67.3%
**Neg. Precision**	89.7%	94.9%
**Neg. Recall**	72.3%	85.8%
**Balanced Acc.**	69.4%	76.6%
**F1 Score**	0.477	0.503
**MCC**	0.324	0.432

Binding site predictor	IDBindT5 with SETH disorder predictions	IDBindT5 with AlphaFold2 disorder predictions	IDBindT5 with experimental disorder annotations
**Precision**	40.9 ± 2.0%	42.1 ± 1.9%	**43.2 ± 1.9%**
**Recall**	39.5 ± 3.0%	42.3 ± 2.8%	**58.7 ± 2.6%**
**Neg. Precision**	66.1 ± 2.4%	66.9 ± 2.6%	**70.2 ± 2.8%**
**Neg. Recall**	**67.3 ± 2.8%**	66.8 ± 2.5%	55.8 ± 2.5%
**Balanced Acc.**	53.4 ± 3.2%	54.5 ± 3.2%	**57.2 ± 3.6%**
**F1 Score**	0.402 ± 0.026	0.422 ± 0.028	**0.498 ± 0.027**
**MCC**	0.069 ± 0.066	0.091 ± 0.065	**0.139 ± 0.070**

* We applied pre-existing disorder predictors to our test set to investigate the impact of using predicted instead of curated disorder information in the input data to IDBindT5. The upper table shows the numeric performance of these disorder predictors on the test set, the lower one shows IDBindT5's performance given these disorder labels. The performance measures were calculated according to the definition in the *Methods* chapter (Eqn. 1-8) and rounded to three decimal points.

Supplementary Table SOM_T6: Performance of disorder predictors on CAID2's binding dataset *

**Model**	**IDBindT5**	**ANCHOR2 [6]**	**DeepDISOBind [7]**	**Random_disorder**
**Residue Subset**	**IDPRs only**	**IDPRs only**	**IDPRs only**	**IDPRs only**
**Precision**	61.4 ± 2.7%	57.0 ± 2.5%	**61.5 ± 3.2%**	55.9 ± 2.5%
**Recall**	69.0 ± 3.6%	60.6 ± 3.2%	**69.4 ± 4.0%**	36.5 ± 1.1%
**Neg. Precision**	51.0 ± 1.9%	43.1 ± 2.4%	**51.3 ± 3.0%**	42.4 ± 2.5%
**Neg. Recall**	42.6 ± 3.8%	39.4 ± 3.1%	42.6 ± 2.4%	**61.9 ± 4.4%**
**Balanced Acc.**	55.8 ± 4.5%	50.0 ± 4.5%	**56.0 ± 4.0%**	49.2 ± 2.7%
**F1 Score**	0.650 ± 0.028	0.587 ± 0.025	**0.652 ± 0.046**	0.441 ± 0.020
**MCC**	0.120 ± 0.086	0.001 ± 0.093	**0.124 ± 0.084**	-0.017 ± 0.024

**Model**	**IDBindT5**	**ANCHOR2**	**DeepDISOBind**	**Random_disorder**
**Residue Subset**	**All**	**All**	**All**	**All**
**Precision**	37.6 ± 2.3%	17.0 ± 3.5%	17.5 ± 3.1%	**57.2 ± 2.5%**
**Recall**	69.0 ± 3.4%	60.6 ± 2.4%	**69.4 ± 2.4%**	37.0 ± 1.6%
**Neg. Precision**	**96.5 ± 3.1%**	94.6 ± 3.1%	95.5 ± 3.4%	43.2 ± 2.4%
**Neg. Recall**	**88.3 ± 2.2%**	69.9 ± 1.4%	66.6 ± 1.3%	63.4 ± 1.2%
**Balanced Acc.**	**78.6 ± 1.6%**	65.2 ± 3.0%	68.0 ± 3.3%	50.2 ± 1.1%
**F1 Score**	**0.487 ± 0.023**	0.266 ± 0.027	0.280 ± 0.034	0.449 ± 0.020
**MCC**	**0.442 ± 0.034**	0.188 ± 0.061	0.217 ± 0.078	0.004 ± 0.020

* Performance assessment on CAID2's [12] binding dataset. The performance was computed for both the IDPR subset (naturally including all positive datapoints), like we did it for our test set in this project, and all residues, like it was done in the CAID challenge [12]. The performance measures were calculated according to the definition in the *Methods* chapter (Eqn. 1-8) and rounded to three decimal points. The number after the ± refers to the 95% confidence interval of the standard error. The numerically best performances per metric and residue subset are printed in bold.

Supplementary Table SOM_T7: Welch's t-test applied to models on the test set. *

**Balanced Accuracy**				
**t-statistic \ p-value**	**IDBindT5**	**Random_disorder**	**ANCHOR2 [6]**	**DeepDISOBind [7]**
**IDBindT5**	\	**0.006**	0.571	0.610
**Random_disorder**	**- 2.790**	\	**0.036**	0.418
**ANCHOR2**	- 0.567	**2.124**	\	0.859
**DeepDISOBind**	- 0.514	0.822	- 0.178	\

**MCC**				
**t-statistic \ p-value**	**IDBindT5**	**Random_disorder**	**ANCHOR2**	**DeepDISOBind**
**IDBindT5**	\	**0.001**	0.854	0.642
**Random_disorder**	**- 3.460**	\	**0.008**	0.290
**ANCHOR2**	- 0.184	**2.729**	\	0.735
**DeepDISOBind**	- 0.469	1.082	- 0.341	\

**Precision**				
**t-statistic \ p-value**	**IDBindT5**	**Random_disorder**	**ANCHOR2**	**DeepDISOBind**
**IDBindT5**	\	0.307	0.897	0.814
**Random_disorder**	- 1.024	\	0.286	0.582
**ANCHOR2**	0.130	1.069	\	0.743
**DeepDISOBind**	- 0.235	0.552	- 0.645	\

**Recall**				
**t-statistic \ p-value**	**IDBindT5**	**Random_disorder**	**ANCHOR2**	**DeepDISOBind**
**IDBindT5**	\	**<0.001**	**0.013**	0.244
**Random_disorder**	**- 5.038**	\	0.210	0.074
**ANCHOR2**	**- 2.508**	1.259	\	0.440
**DeepDISOBind**	- 1.171	1.817	0.774	\

* The scipy implementation of Welch's t-test [13, 14] was applied to different metrics of all models on the test set. In contrast to the student's t-test it does not assume equal population variance. If the p-value (upper-right triangles) is smaller than 0.05, then we have evidence against the null hypothesis of equal population means [13], so there is a

significant difference (printed in bold). The t-statistic (lower-left triangles) quantifies the difference between the arithmetic means of the rows's model vs. the columns's model. All values are rounded to three decimal points.

Supplementary Table SOM_T8: Speed assessment *

**Conditions**			**Predictor (median runtime)**
**Dataset**	**Machine**	**GPU Usage**	**IDBindT5**
P09938 (399 AAs)	Consumer-grade	No	2.246 s
P09938 (399 AAs)	Consumer-grade	Yes	2.378 s
Test set (Mobi195)	Consumer-grade	No	12.594 s
Test set (Mobi195)	Consumer-grade	Yes	8.146 s

**Conditions**			**Predictor (absolute runtime)**		
**Dataset**	**Machine**	**GPU Usage**	**IDBindT5**	**ANCHOR2 [6]**	**DeepDISOBind [7]**
Mobi11k	Cluster	Yes	2 h, 10 min	21 min	33 h, 22 min

* Speed assessment under different conditions (dataset, machine, usage of GPU). We provide the median runtime of 10 single prediction runs on the small datasets (upper table) per condition, and the absolute runtimes on the biggest dataset (lower table). The sequence of the protein P09938 (Ribonucleoside-diphosphate reductase small chain 1) has a sequence length of 399, which matches the median sequence length of our training set (Mobi2k). The consumer-grade machine's stats are: CPU: Intel Core i7, 16 GB RAM, 4 cores; GPU: NVIDIA GeForce MX350, 9.9 GB vRAM. The cluster's stats are: CPU: 2x Intel Xeon Gold 6248, 400 GB RAM, 20 cores; GPU: 4x Quadro RTX 8000, 46 GB vRAM.

References for Supporting Online Material

1 Lin, Z. *et al.* Language models of protein sequences at the scale of evolution enable accurate structure prediction. *bioRxiv*, 2022.2007.2020.500902 (2022). https://doi.org:10.1101/2022.07.20.500902

2 Littmann, M., Heinzinger, M., Dallago, C., Weissenow, K. & Rost, B. Protein embeddings and deep learning predict binding residues for various ligand classes. *Sci Rep* **11**, 23916 (2021). https://doi.org:10.1038/s41598-021-03431-4

3 Bernhofer, M. & Rost, B. TMbed: transmembrane proteins predicted through language model embeddings. *BMC Bioinformatics* **23**, 326 (2022). https://doi.org:10.1186/s12859-022-04873-x

4 Marquet, C. *et al.* Embeddings from protein language models predict conservation and variant effects. *Hum Genet* **141**, 1629-1647 (2022). https://doi.org:10.1007/s00439-021-02411-y

5 Weissenow, K., Heinzinger, M. & Rost, B. Protein language-model embeddings for fast, accurate, and alignment-free protein structure prediction. *Structure* **30**, 1169-1177.e1164 (2022). https://doi.org:10.1016/j.str.2022.05.001

6 Mészáros, B., Erdos, G. & Dosztányi, Z. IUPred2A: context-dependent prediction of protein disorder as a function of redox state and protein binding. *Nucleic Acids Res* **46**, W329-W337 (2018). https://doi.org:10.1093/nar/gky384

7 Zhang, F., Zhao, B., Shi, W., Li, M. & Kurgan, L. DeepDISOBind: accurate prediction of RNA-, DNA- and protein-binding intrinsically disordered residues with deep multi-task learning. *Brief Bioinform* **23** (2022). https://doi.org:10.1093/bib/bbab521

8 Elnaggar, A. *et al.* ProtTrans: Towards Cracking the Language of Life's Code Through Self-Supervised Deep Learning and High Performance Computing. *- under review -* (2021). https://doi.org:10.1101/2020.07.12.199554

9 Kawashima, S. *et al.* AAindex: amino acid index database, progress report 2008. *Nucleic Acids Res* **36**, D202-205 (2008). https://doi.org:10.1093/nar/gkm998

10 Ilzhöfer, D., Heinzinger, M. & Rost, B. SETH predicts nuances of residue disorder from protein embeddings. *Frontiers in Bioinformatics* **2** (2022). https://doi.org:10.3389/fbinf.2022.1019597

11 Piovesan, D., Monzon, A. M. & Tosatto, S. C. E. Intrinsic protein disorder and conditional folding in AlphaFoldDB. *Protein Sci* **31**, e4466 (2022). https://doi.org:10.1002/pro.4466

12 Conte, A. D. *et al.* Critical assessment of protein intrinsic disorder prediction (CAID) - Results of round 2. *Proteins* (2023). https://doi.org:10.1002/prot.26582

13 Virtanen, P. *et al.* SciPy 1.0: fundamental algorithms for scientific computing in Python. *Nat Methods* **17**, 261-272 (2020). https://doi.org:10.1038/s41592-019-0686-2

14 Welch, B. L. The generalisation of student's problems when several different population variances are involved. *Biometrika* **34**, 28-35 (1947). https://doi.org:10.1093/biomet/34.1-2.28
